# Thyroid hormones derivatives reduce proliferation and induce cell death and DNA damage in ovarian cancer

**DOI:** 10.1038/s41598-017-16593-x

**Published:** 2017-11-28

**Authors:** Elena Shinderman-Maman, Keren Cohen, Dotan Moskovich, Aleck Hercbergs, Haim Werner, Paul J. Davis, Martin Ellis, Osnat Ashur-Fabian

**Affiliations:** 1 Translational Hemato-Oncology Laboratory, The Hematology Institute and Blood Bank, Meir Medical Center, Kfar-Saba, Israel; 2 Department of Human Molecular Genetics and Biochemistry, Tel Aviv University, Tel Aviv, Israel; 3 Sackler Faculty of Medicine, Tel Aviv University, Tel Aviv, Israel; 4 Radiation Oncology, Cleveland Clinic, Cleveland, OH USA; 5 Department of Medicine, Albany Medical College, Albany, NY USA

## Abstract

Ovarian cancer is a highly aggressive disease and novel treatments are required. Thyroid hormones binding to αvβ3 integrin produced growth-promoting activities in ovarian cancer and we hypothesized that natural thyroid hormone derivatives may antagonize these actions. The effect of three antagonists, tetraiodoacetic acid (tetrac), triiodothyroacetic acid (triac) and 3-iodothyronamine (T1AM), on cell proliferation, cell death and DNA damage was studied in two ovarian cancer cell lines (OVCAR3 and A2780), normal hamster ovary control cells (CHOK1) and αvβ3-deficient or transfected HEK293 cells. A differential inhibition of cell proliferation was observed in ovarian cancer cells compared to CHOK1. In OVCAR3, an induction of cell cycle regulators was further shown. Apoptosis was confirmed (annexin-PI, SubG1/cell-cycle, apoptotic genes, caspase-3 and poly ADP ribose polymerase-1 (PARP-1) cleavage) and was reversed by a pan-caspase inhibitor. Induction in apoptosis inducing factor (AIF) was observed, suggesting a parallel caspase-independent mechanism. Integrin-involvement in triac/T1AM apoptotic action was shown in αvβ3-transfected HEK293 cells. Lastly, in ovarian cancer models, key proteins that coordinate recognition of DNA damage, ataxia-telangiectasia mutated (ATM) and PARP-1, were induced. To conclude, the cytotoxic potential of thyroid hormone derivatives, tetrac, triac and T1AM, in ovarian cancer may provide a much-needed novel therapeutic approach.

## Introduction

Ovarian cancer is a highly metastatic disease and the second leading cause of death from gynecologic malignancies^1,2^. The 5-year survival rate of women diagnosed with late stage ovarian cancer is less than 50%^3,4^ and novel treatments are needed.

Thyroid hormones have been shown to demonstrate non-genomic activity in addition to their classical genomic action (Reviewed in^5–7^). The former is mediated via alternate pathways, not involving binding of the biological hormone triiodothyronine (T3) to its nuclear thyroid receptors (TRα or TRβ) and are initiated by both T3 and its pro-hormone thyroxine (T4). One of the mechanisms whereby such non-genomic actions may be mediated is via binding of T3 and T4 to the extracellular domain of integrin αvβ3^8^. This axis is highly relevant in oncology since this integrin is overexpressed in an array of cancer types and correlates with disease stage^9^. Binding of both hormones is specific for αvβ3; that is, they do not bind to other integrins^10^. Upon binding, T3 and T4 produce diverse non-genomic, membrane-initiated activities (Reviewed in^7^), including proliferative effects mainly via the MAPK pathway. Such mitogenic activities were shown in various types of cancer cells including glioma^11^, breast cancer^12^, hepatocarcinoma^13^, thyroid cancer^14^, sarcoma^15^ and tumor-associated vascular cells^16^. We have demonstrated similar growth-promoting effects of thyroid hormones, via direct binding to the αvβ3 integrin, in myeloma^17–19^ melanoma^20^ and ovarian cancer^21^. Based on these collective results we hypothesized that natural thyroid hormone derivatives with low-potency thyromimetic activity may produce an opposite effect on the integrin binding site which may be utilized for growth inhibition in ovarian cancer cell models.

Such analogues include a deaminated form of thyroxine, tetraiodoacetic acid (tetrac), a deaminated form of triiodothyronine, triiodothyroacetic acid (triac) and 3-iodothyronamine (T1AM), a deiodinated derivative of thyroxine. Tetrac and triac possess low hormone activity because of shortening of the side chain on the inner ring (removal of a carbon or amine), resulting in the conversion of propionic acid (thyroid hormone) to acetic acid (tetrac/triac). This transforms the compounds from thyroid agonists to antagonists^6^. T1AM has no affinity for TRα or TRβ, or an ability to stimulate or inhibit nuclear TR–mediated transactivation^22^. Therefore, the hormone derivatives used by us, have little or no affinity to the nuclear thyroid hormone receptors, through which the classical genomic actions are initiated by the thyroid hormone and are expected to possess only antagonistic actions at the cell surface integrin receptor (displacement of T4/T3). We herein report the effect of these antagonists on reducing proliferation and induction of apoptosis and DNA damage response in ovarian cancer cell lines.

## Results

### Thyroid hormone derivatives affect ovarian cancer cell proliferation and viability

**Figure 1:**
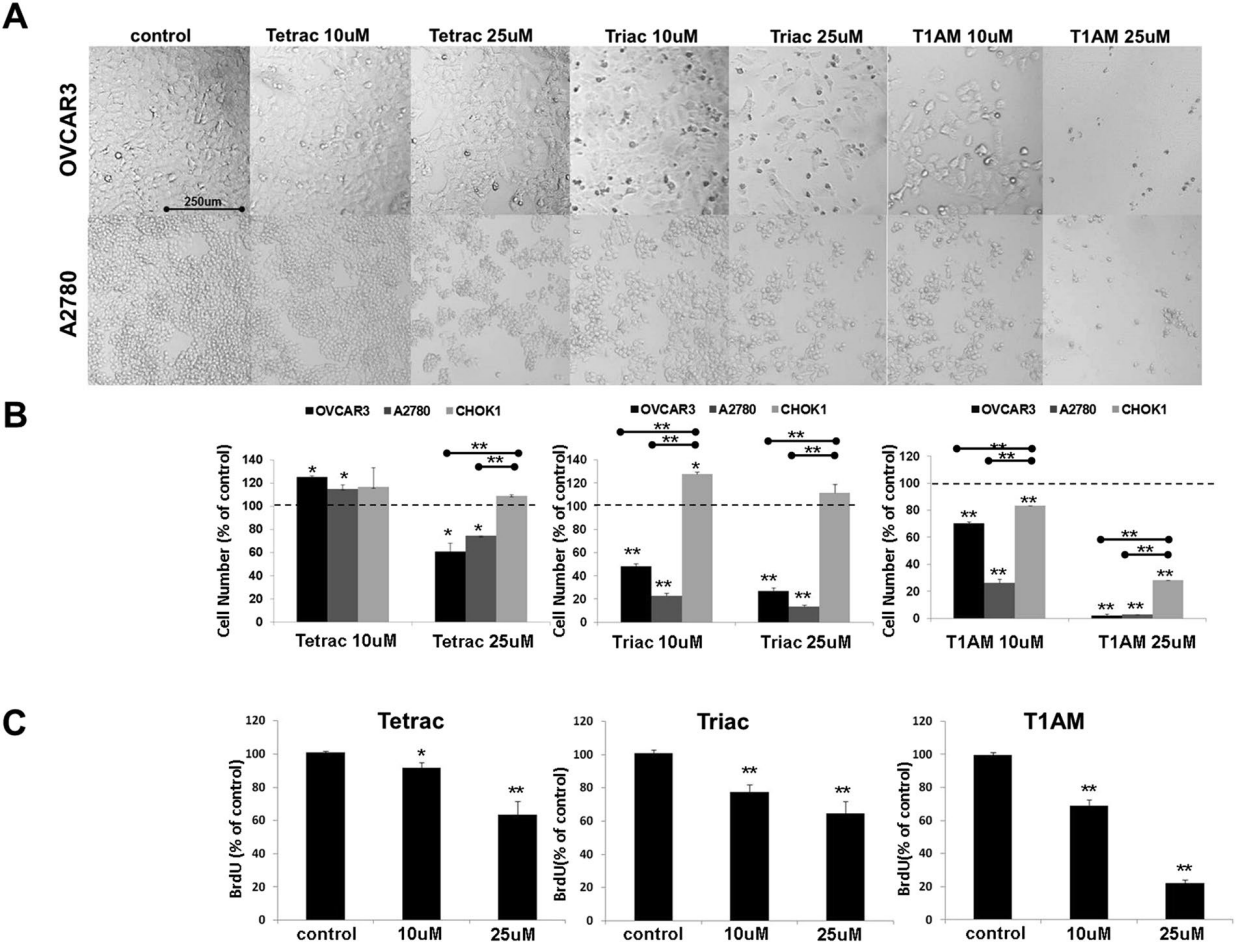
Tetrac, triac and T1AM affect cell proliferation and viability. Cells were treated with the analogs (10 or 25 μM) for 96 hours and assessed by (**A**) light microscopy (**B**) absolute cell number (flow-cytometry) (**C**) proliferation (BrdU, ELISA). Experiments were repeated 3 times in triplicates. Significance (*p < 0.05, **p < 0.005) from vehicle control (KOH) or from normal control cells (indicated by ) is shown.

The effect of tetrac, triac and T1AM on cancer cell proliferation and viability was examined *in vitro* in two ovarian cancer cell lines, OVCAR3, with high αvβ3 integrin levels and A2780, with lower integrin expression. These cells were shown by genomic profiling to represent high-grade serous^23^ and low-grade endometrioid^23–26^ disease, respectively. In addition, normal ovarian cells (CHOK1), which hardly express αvβ3 on their cell surface (Supplemental Fig. 1), were used as negative control. The cells (10,000 cells/96-well plates) were treated daily with the three analogues at 1, 10 or 25 μM concentrations for 24–96 hours and analyzed for proliferation and viability by several methods. The effect at the differing concentrations and exposure time (24–96 hours) is depicted in Supplementary Fig. 2. Representative images of 10 and 25 μM treatments for 96 hours, in which a maximal effect was documented, are shown in Fig. 1A. Cell density was reduced in both cell lines after treatment with tetrac at 25 μM, while a dose-dependent effect was shown for triac and T1AM at 10 and 25μM. Absolute cell counts (cells/μl) using flow-cytometry validated these results (Fig. 1B). In details, concordant with the microscopy results, following 96 hours with 25 μM of tetrac (Fig. 1B, Left panel) a 30–40% reduction in cell number, in comparison to vehicle control, was clearly observed in both ovarian cancer cell lines. At lower tetrac concentrations (10 μM and below), an agonistic effect on cell number was demonstrated (Supplementary Fig. 2A). Triac (Fig. 1B, middle panel) and T1AM (Fig. 1B, right panel), had a potent inhibitory effect on OVCAR3 and A2780, at both examined concentrations. In normal CHOK1 cells no inhibitory effect by tetrac or triac was observed, while T1AM produced a lower effect compared to OVCAR3 and A2780.

**Figure 2:**
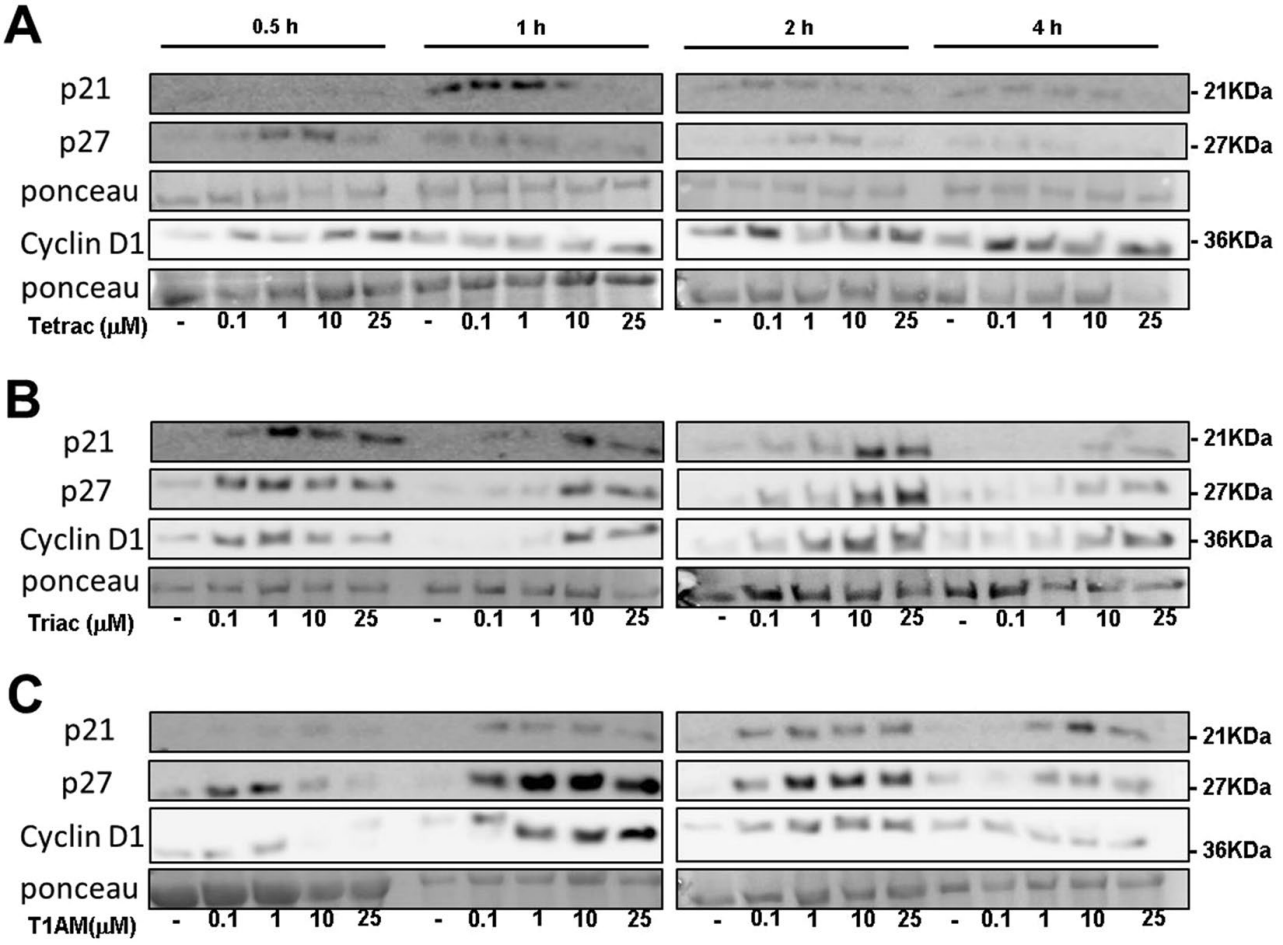
Thyroid hormone derivatives induce cell cycle regulators. OVCAR3 cells were treated for 0.5–4 hours with 0.1, 1, 10 or 25 μM (**A**) tetrac (**B**) triac and (**C**) T1AM, respectively. Protein levels of p21, p27 and cyclin D1 were analyzed by Western blots. Representative blot of two independent experiments is presented.

To investigated whether the reduction in cell number is due to a direct effect on cell proliferation, DNA synthesis was measured (BrdU assay). In OVCAR3 cells (Fig. 1C) a significant dose-dependent reduction in DNA synthesis was observed by the three analogues, with T1AM being the most potent inhibitor. We have further evaluated by Western blots the level of three cell cycle regulators, p21, p27 and cyclin D1 in OVCAR3 cells (100,000 cells/24-well plates) treated with tetrac, triac or T1AM (0.1, 1, 10 and 25 μM) for 0.5–4 hours. Total proteins were extracted to perform Western blot analysis. Representative blots are presented in Fig. 2 and normalized optical densities are presented in Supplementary Fig. 3. In details, results indicate that tetrac (Fig. 2A) induced the protein level of p21, peaking at 1 and 2 hours of treatment, while for p27 and Cyclin D1 the highest effect was observed as early as 0.5 hours and again following 2 hours of treatment. Triac (Fig. 2B) upregulated p21, p27 and cyclin D1 after 0.5 hours of treatment, an effect which was continued up to 4 hours. Results for T1AM (Fig. 2C) indicated an induction in p21 at 1–4 hours of treatment, while p27 was induced at 0.5 hours, sustaining up to 4 hours of treatment. The effect of T1AM on cyclin D1 induction was observed between 1-2 hours of treatment and was diminished after 4 hours. Collectively, the results presented above suggest that the mechanism of action by the antagonists involves inhibition of ovarian cancer cell proliferation.

**Figure 3:**
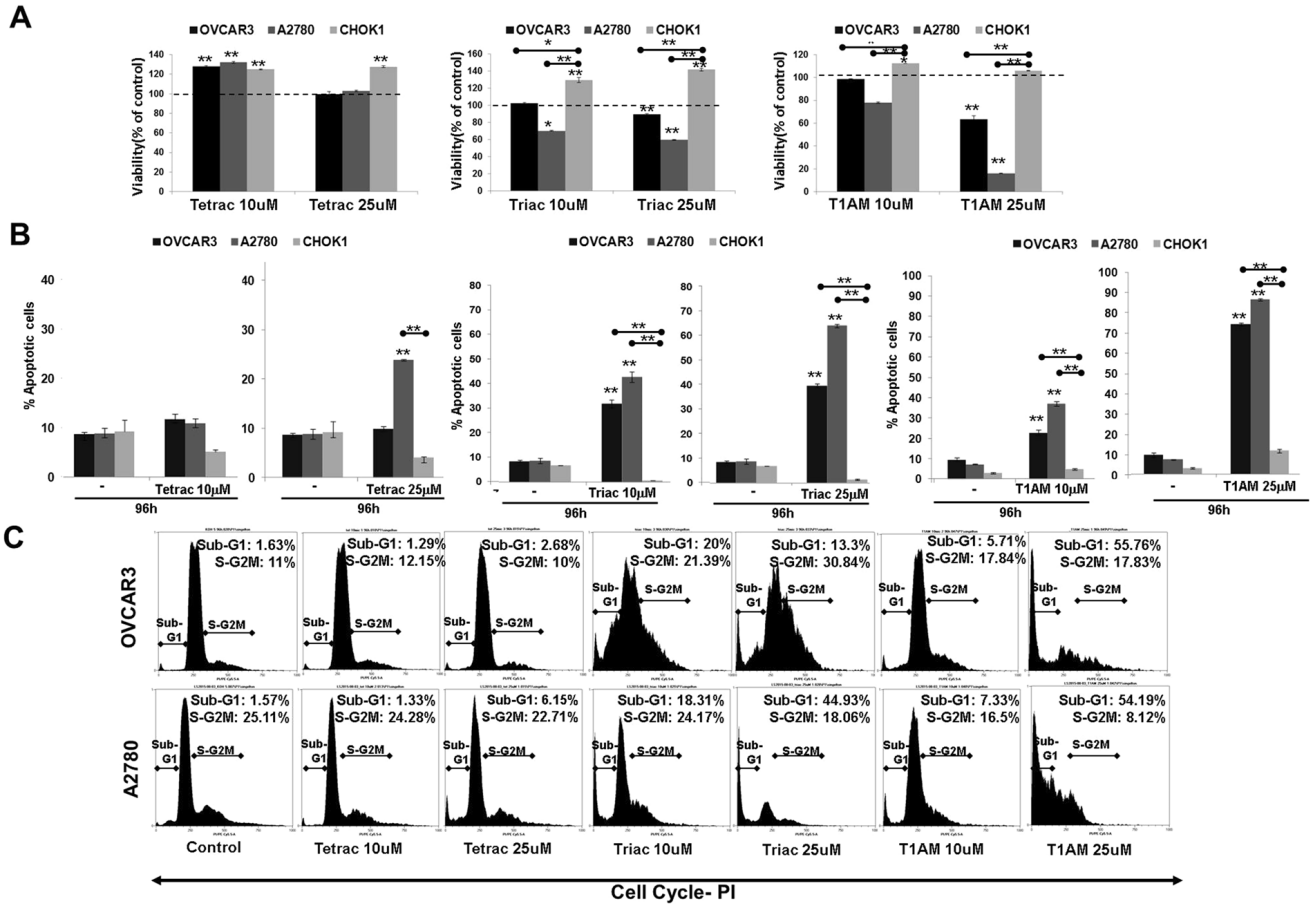
Tetrac, triac and T1AM induce apoptosis. Cells were treated with the analogs (10 or 25 μM) for 96 hours and assessed by (**A**) Cell viability (PrestoBlue) (**B**) annexin-FITC/PI and (**C**) Cell cycle distribution. Experiments were repeated 3 times in triplicates. Significance (*p < 0.05, **p < 0.005) from vehicle control (KOH) or from normal control cells (indicated by ) is shown.

Next, we studied whether the antagonists’ effect is restricted to cell proliferation or is accompanied by an effect on cell viability and death. Results indicate that no reduction in cell viability was observed by tetrac (Fig. 3A), while triac and T1AM inhibited cell viability in OVCAR3 at 25 μM and at both concentrations in A2780. Cell viability was increased by tetrac/triac/T1AM at 10 and 25 μM in the normal control cells, while in OVCAR3 and A2780 a similar increase was shown by tetrac at 10 μM.

After observing an effect on viability, we studied the analogues effect on cell death (annexin-V/PI, flow cytometry). OVCAR3, A2780 and CHOK1 cells (10,000 cells/96-well plates) were treated with tetrac, triac or T1AM (1, 10 or 25 μM) for 24–96 hours. The percentage of apoptotic cells (early apoptosis (An+/PI−) and late apoptosis/necrosis (An+/PI+, An−/PI+, respectively)) is presented in Supplementary Fig. 4. The effects of the three agents (10–25 μM) at the longest incubation time (96 hours) are depicted in Fig. 3B. Specifically, tetrac induced apoptosis at 25 μM only in A2780 cells, with no apparent effect on OVCAR3. Triac and T1AM potently induced apoptosis in the two cell models at both examined concentrations. No effect was observed in the normal control cells. Representative flow-cytometry analysis for OVCAR3 and A2780 are presented in Supplementary Fig. 5.

Next we assessed the analogues effects on the different phases of cell cycle in the two ovarian cancer models compared to CHOK1 cells. A representative cell cycle analysis for OVCAR3 and A2780 is depicted in Fig. 3C. No significant effect on cell cycle was observed by tetrac, while treatment with triac or T1AM produced an effect that was analogue-specific. In details, triac produced a differential effect in the two ovarian cancer cell lines. In OVCAR3 cells triac produced, in parallel to apoptosis (represented by the subG1 cell fraction) a potent S-G2M arrest, while in A2780 cells, triac exclusively induced SubG1. For T1AM, apoptosis was observed in both ovarian cancer cell lines, mainly at 25 μM concentration. The complete SubG1 data is depicted in Supplementary Fig. 6, indicating a minimal or no effect in the normal control cells.

**Figure 4:**
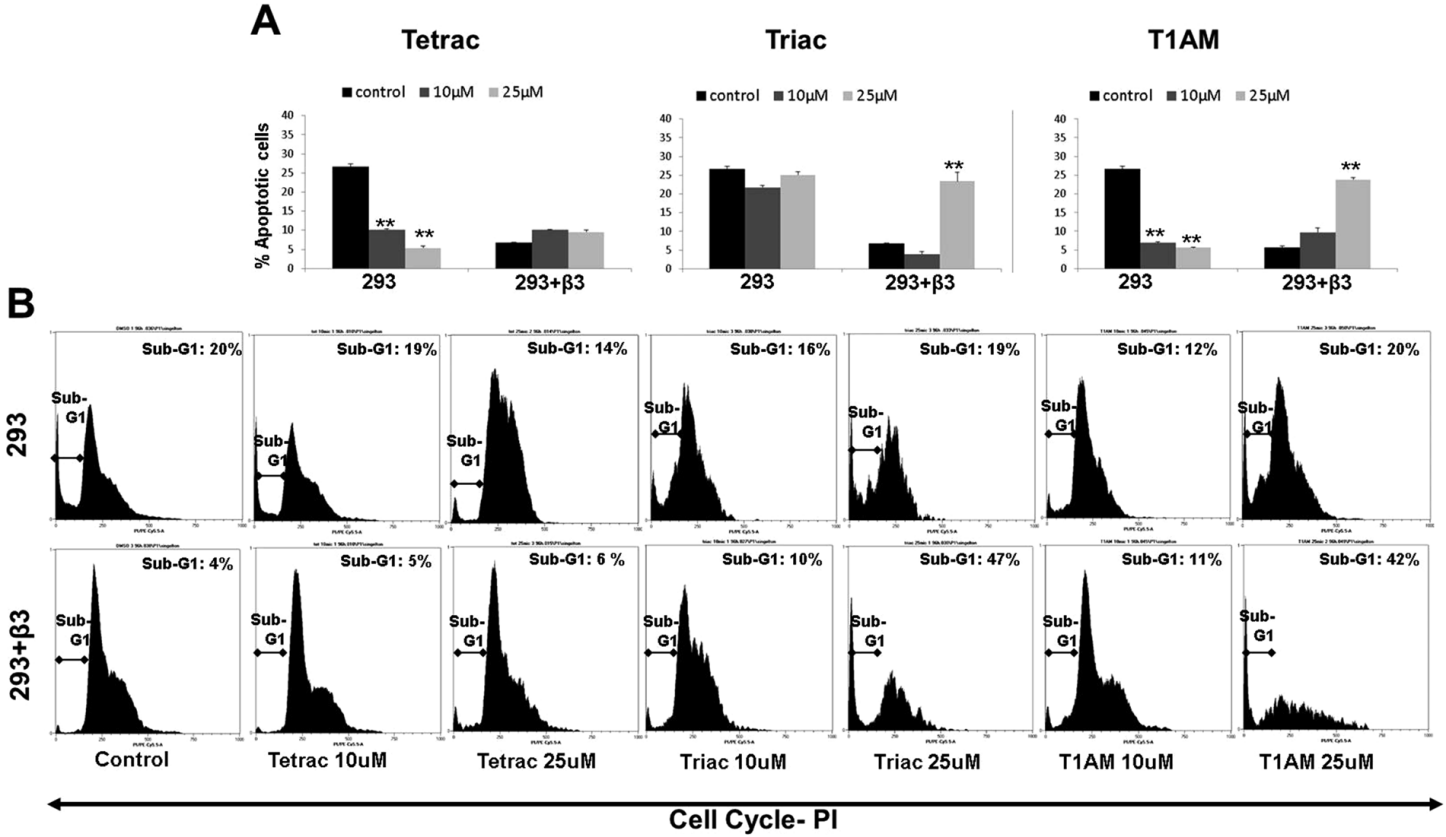
Triac and T1AM induce apoptosis in αvβ3-transfected HEK-293 cells. HEK-293 cells lacking or overexpressing αvβ3 integrin were treated in triplicates with 10 or 25 μM tetrac, triac or T1AM for 96 hours and analyzed for (**A**) annexin-FITC/PI and (**B**) cell cycle distribution. Significance (*p < 0.05, **p < 0.005) from vehicle control (KOH) is shown.

Taken together, the effects of the three analogues on cell proliferation, viability and cell cycle distribution were analogue and-cell-type-specific. Lastly, we have utilized HEK293 cells lacking αvβ3 expression or transfected with this receptor to study the hormone-derivative cytotoxic action. Using this cellular platform we were able to demonstrate that the effect of 25 μM triac and T1AM on apoptosis (annexin-PI, Fig. 4A) and cell cycle (Fig. 4B) is dependent on αvβ3 integrin expression.

### Thyroid hormone analogues induce caspase-mediated apoptosis and AIF levels

**Figure 5:**
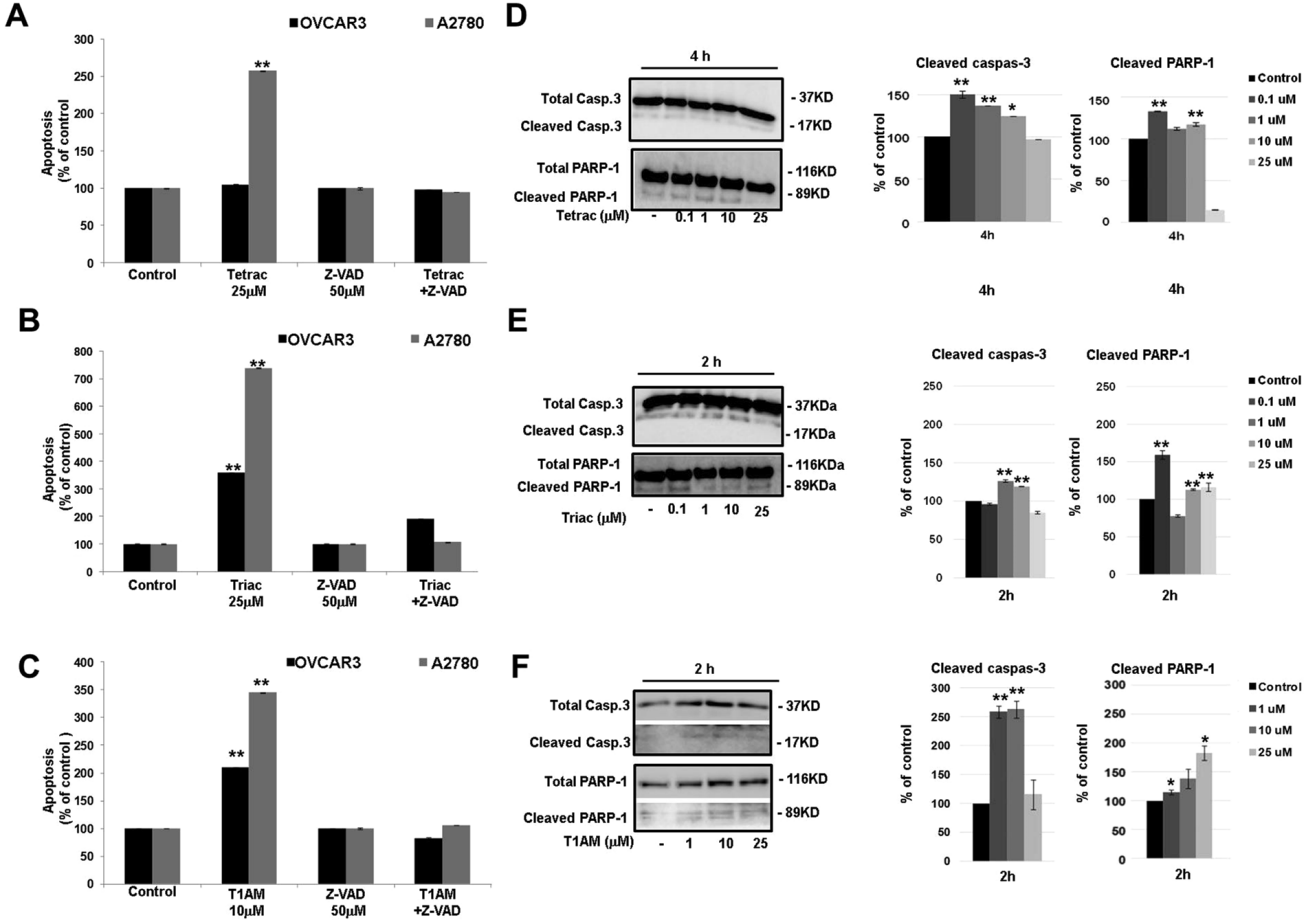
Caspase-dependent apoptosis by tetrac, triac and T1AM. Cells were treated with 25 μM (**A**) tetrac (**B**) triac (**C**) 10 μM T1AM, for 96 hours ± Z-VAD-FMK (50 μM) and analyzed by annexin-V/PI. Experiments were repeated twice in triplicates. Cells were treated with 0.1, 1, 10 or 25 μM (**D**) tetrac (**E**) triac or (**F**) T1AM, for 2 or 4 hours and caspase-3 and PARP-1 cleavage were  analyzed by Western blots. Representative blot (cropped) is presented. Experiments were repeated twice. Significance from vehicle control (KOH) is indicated by *p < 0.05, **p < 0.005.

Next we examined whether the induction in apoptosis produced by tetrac, triac and T1AM, is caspase-mediated. OVCAR3 and A2780 cells (10,000 cells/96-well plate) were incubated with tetrac, triac (25 μM) or T1AM (10 μM) for 96 hours in the presence or absence of the pan-caspase inhibitor, Z-VAD-FMK (50 μM). The increase in apoptosis (annexin-V/PI, flow cytometry) by tetrac was completely abrogated in the presence of Z-VAD-FMK in the low-grade A2780 cells (Fig. 5A), indicating caspases involvement. Comparable results were obtained for triac (Fig. 5B) and for T1AM (Fig. 5C) in both cell models. Of notice, in OVCAR3 cells triac effect was only partly attenuated by Z-VAD-FMK, suggesting that in these cells the effect is not exclusively caspase-dependent. Representative annexin-V/PI results for the three analogues in the presence of Z-VAD-FMK are depicted in Supplemental Fig. 7. Overall, these collective results imply that tetrac, triac and T1AM induce apoptosis that is primarily caspase-mediated.

To explore whether caspase-3 is the effector caspase that is involved in the death induced by the three analogues, the level of the procaspase-3 (37 kDa) and its active/cleaved form (17 kDa) were analyzed. Additionally, the cleavage of a downstream substrate of caspase-3, poly ADP ribose polymerase-1 (PARP-1, 89 kDa), which is considered to be a biomarker for apoptosis, was evaluated. Both OVCAR3 and A2780 cells (100,000 cells/24-well plates) were treated with tetrac, triac or T1AM at increasing concentrations (100 nM-25 μM) and increasing incubation times (0.5–4 h) and total proteins were extracted to perform Western blots analysis. Supplemental Fig. 8 depicts the entire time and concentration range and indicates no cleavage in caspase-3 or PARP-1 in the high-grade OVCAR3 cells by the three analogues, suggesting the involvement of an alternate effector caspase. However, in A2780 cells, in accordance with the annexin-PI experiments, treatment with tetrac induced caspase-3 activation (24–50%, normalized to total caspase-3) and PARP-1 cleavage (17–32%, normalized to total PARP-1) at 4 hours of treatment (Fig. 5D). Triac (1–10 μM) induced caspase-3 activation by about 20%, after 2 hours of treatment, followed by a parallel PARP-1 cleavage (Fig. 5E). T1AM produced an elevation in caspase-3 activation (150%) after 2 hours of treatment (Fig. 5F) that was accompanied by an elevation of 15–82% in the levels of cleaved PARP-1.

**Figure 6:**
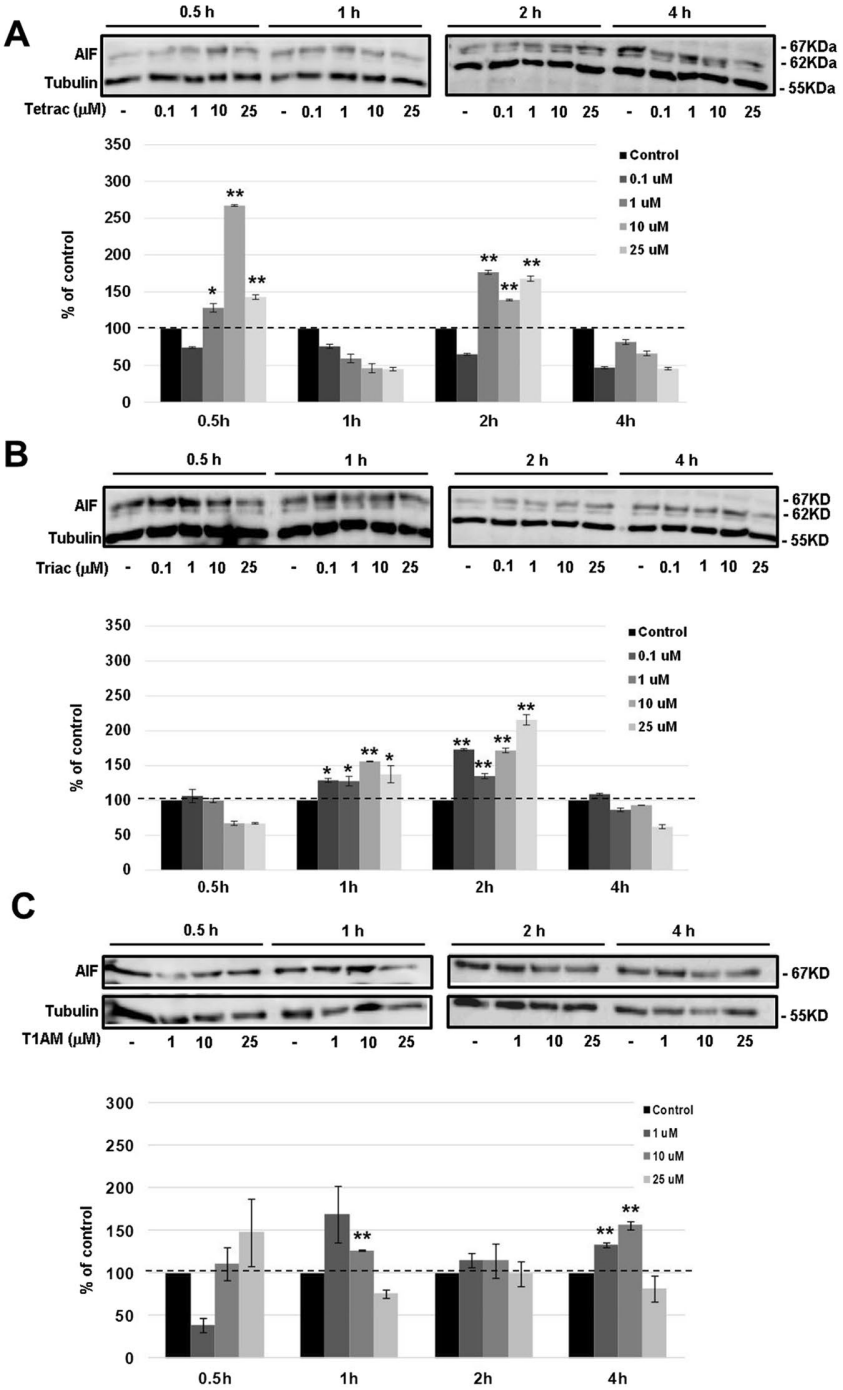
Tetrac, triac and T1AM induce AIF. Cells were treated with 0.1, 1, 10 or 25 μM (**A**) tetrac (**B**) triac (**C**) T1AM for 0.5–4 hours and AIF levels were evaluated by Western blots. Representative blot of two independent experiments is presented. *p < 0.05, **p < 0.005.

We then explored the involvement of caspase-independent apoptosis by measuring the protein level and activation of apoptosis-inducing factor (AIF). This 67 kDa protein is cleaved by peptidase generating the mature 62 kDa protein. In A2780 cells (100,000 cells/24-well plates) AIF protein was clearly induced by tetrac (Fig. 6A), triac (Fig. 6B) and T1AM (Fig. 6C), suggesting a mechanism involving both caspase-dependent and independent apoptosis. For OVCAR3 cells the results, presented in Supplemental Fig. 9 indicate that AIF is barely detected and was not induced by the three thyroid hormone analogues.

### Apoptosis-relevant gene expression in response to hormone analogues in ovarian cancer

**Figure 7:**
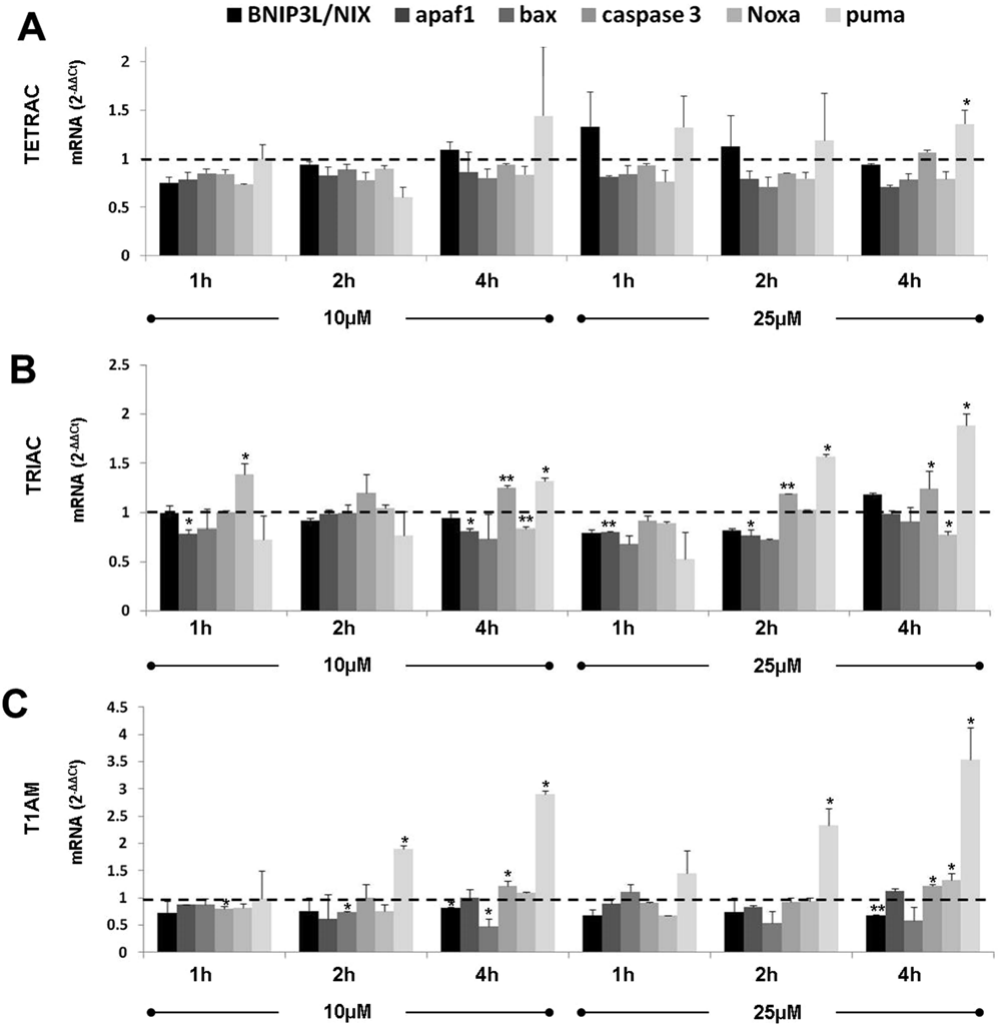
Tetrac, triac and T1AM affect apoptotic gene expression. Cells were treated for 1–4 h with 10 or 25 μM of (**A**) tetrac, (**B**) triac (**C**) T1AM and mRNA levels were assessed by real-time PCR. Results were calculated as fold change using the comparative threshold cycle method (2-ΔΔCT) relative to control cells (that is, controls are arbitrarily assigned a value of 1). Experiments were repeated three times and are presented (average ± STDEV) as fold of control (1 ± STDEV, dashed line). *p < 0.05, **<0.005.

We subsequently studied the effect of tetrac, triac and T1AM on the transcription of a number of apoptosis-relevant genes. OVCAR3 cells (100,000 cells/24-well plates) were treated with 10 µM or 25 µM concentration of the analogues and RNA was extracted at three time points (1, 2 and 4 hours). Results show that each agent caused a different profile of gene expression, in terms of induction, down-regulation or absence of action. In details, for tetrac, the only apoptotic gene that was significantly induced was puma, with 25 μM concentration following 4 hours of incubation (Fig. 7A). Treatment of OVCAR3 cells with triac (Fig. 7B) resulted in a differential effect, with an induction in *caspase-3* (18–33% increase), *noxa* (40% increase) and *puma* (32–88% increase), while *apaf-1* was inhibited (20% decrease). Lastly, incubation of the cells with T1AM (Fig. 7C) resulted in an induction in the mRNA levels of *caspase-3* (20% increase), *noxa* (33% increase), with a pronounced increase in *puma* (200–350% increase). Taken together, different sets of apoptotic genes were affected, at a dose and time that was agent-specific.

### Induction of DNA-damage markers by thyroid hormone derivatives in ovarian cancer

**Figure 8:**
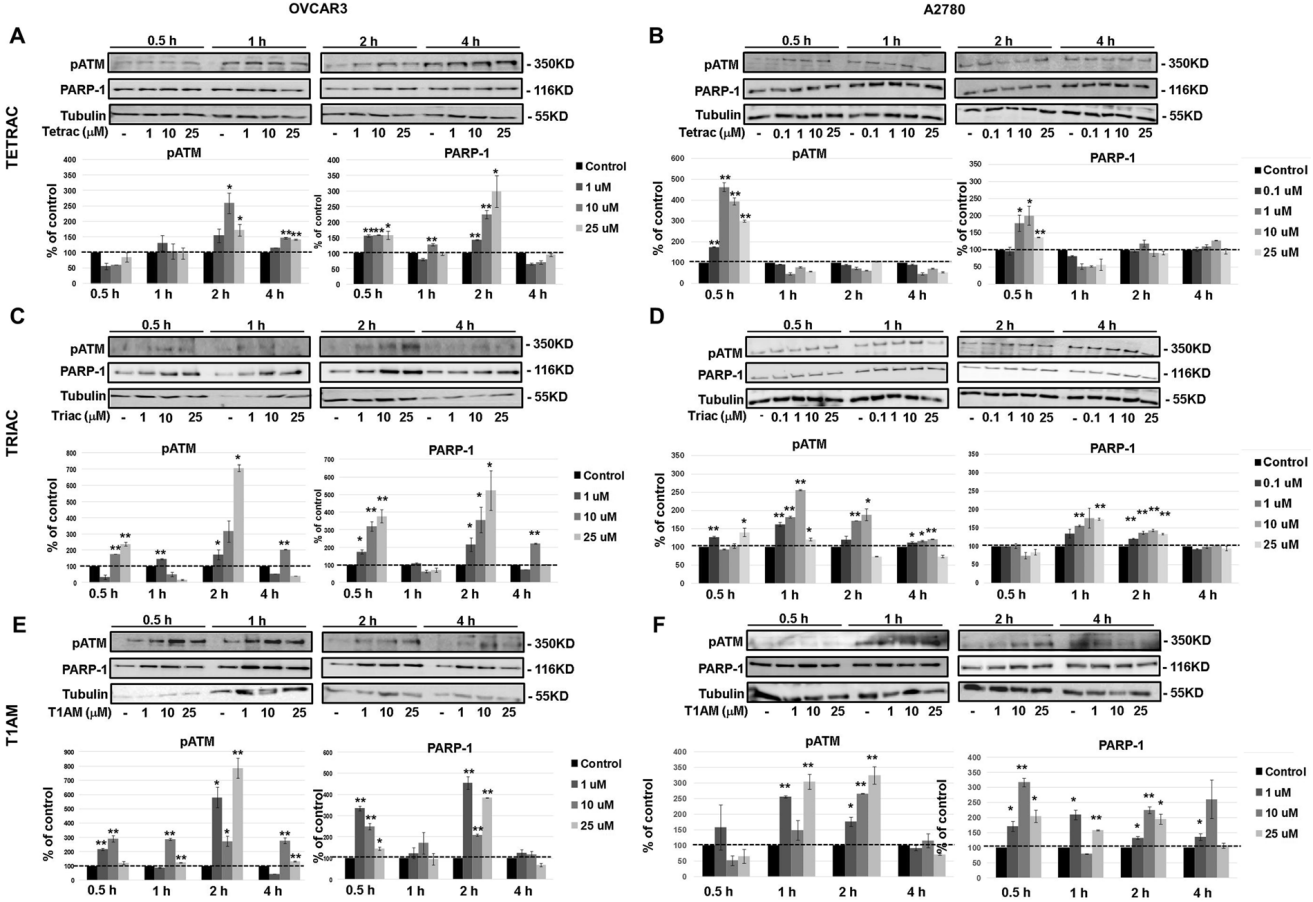
Thyroid hormone derivatives induce ATM phosphorylation and PARP-1. OVCAR3 and A2780 cells were treated for 0.5–4 hours with 0.1, 1, 10 or 25 μM (**A**,**B**) tetrac (**C**,**D**) triac and (**E**,**F**) T1AM, respectively. ATM and PARP-1 were analyzed by Western blots. Representative blot of two independent experiments is presented. *p < 0.05, **p < 0.005.

Many cytotoxic antagonists lead to cell death via their effect on DNA damage and repair processes. We have therefore evaluated the level of two key markers for DNA damage response, phosphorylated ataxia-telangiectasia mutated (ATM) and PARP-1. Both ovarian cancer cell models (100,000 cells/24-well plates) were treated with tetrac, triac or T1AM (0.1–25 μM/A2780 and 1–25 μM/OVCAR3 cells) for 0.5–4 hours and protein levels were assessed by Western blots. Results indicate that the three agents induced ATM phosphorylation and PARP-1 protein in both OVCAR3 and A2780 cells, at concentrations and time range that were cell-specific (Fig. 8). Specifically, tetrac in OVCAR3 (Fig. 8A) significantly increased ATM phosphorylation and PARP-1 protein levels by up to 2.5-fold and 3-fold, respectively, after 2 hours. In A2780 cells (Fig. 8B) an induction of about 4.5-fold in ATM phosphorylation and 3-fold in PARP-1 was shown following incubation with tetrac for 0.5 hours. Regarding the effect of triac on DNA damage response markers, results indicate that OVCAR3 cells (Fig. 8C) were more likely to increase DNA damage markers in response to triac (6-7-fold increase), in comparison to 1.8-2.5-fold increase in A2780 (Fig. 8D).

Similarly, the effect of T1AM on DNA damage/response markers was more pronounced in OVCAR3 cells peaking to 8-fold for ATM phosphorylation and 4.5-fold for PARP-1 (Fig. 8E) compared to a 3-fold increase in A2780 cells (Fig. 8F).

After observing that the three thyroid hormone derivatives lead to an elevation in DNA damage markers in our cell models, we were interested to explore whether these agents can directly interact with the DNA molecule and lead to DNA breaks. To this end, a cell-free experimental platform was utilized (detailed in the methods section). Linear DNA ladder, as well as DNA that was extracted from OVCAR3 cells, were examined. Both DNA samples were incubated overnight in the presence or absence of a high dose of tetrac, triac or T1AM (50 μM) and were separated by electrophoresis. No effect on DNA migration or DNA breaks was evident in neither sample (Supplemental Fig. 10), suggesting that the DNA-damage response by the three analogues was indirect.

## Discussion

In this study we examined the ability of tetrac, triac and T1AM, natural thyroid-hormones derivatives, to inhibit the growth and viability of ovarian cancer cells. Tetrac and triac possess low hormone activity because of shortening of the side chain on the inner ring (removal of a carbon or amine), resulting in the conversion of propionic acid (thyroid hormone) to acetic acid (tetrac/triac). This transforms the compounds from thyroid agonists to antagonists^6^. Tetrac binds the αvβ3 integrin at two orientations and competitively displaces both T3 and T4 binding and thus inhibits their activity^8,27^. Antagonistic effects of tetrac were studied in several *in-vitro* and animal cancer models (reviewed in^7^). These included renal cell carcinoma^28^, medullary/follicular thyroid cancer^29,30^, chemo-resistant breast cancer^31,32^ and pancreatic cancer^33^. However to date this compound has not been examined in ovarian cancer. Triac, like tetrac, is capable of blocking non-genomic actions of thyroid hormones^34–36^. However, these activities were demonstrated so far in non-malignant cell lines^35^ and the only cancer model in which it has been partly implicated is cervical cancer^36^. The third antagonist that was examined is T1AM, a deiodinated T4 derivative^37^. There is no data available regarding integrin binding or blocking by T1AM and, to the best of our knowledge, this derivative has never been studied in cancer cells in general and ovarian cancer in particular. Our data from integrin transfected cells, implies that this derivative also exert its antagonistic action via αvβ3.

The effect of the three thyroid hormone derivatives was examined *in vitro* in two ovarian cancer cell lines which were recently shown by genomic profiling^23^ to represent a high-grade serous ovarian cancer (OVCAR3) and low-grade disease (A2780, endometroid subtype^23–26^). Both cell lines express αvβ3 integrin, with a higher expression level in OVCAR3^21^. These findings concur with reports of the high expression levels of this integrin in pathological tissues obtained from high-grade serous ovarian cancer patients, and low levels in low-grade cases^38,39^. Consequently, the cell models we used are clinically relevant. In addition, the use of normal ovarian cells (CHOK1), which hardly express membrane αvβ3 integrin, served as normal control.

Our current results indicate that while the three antagonists did not affect CHOK1 normal control cells, proliferation rate of ovarian cancer cells was effectively reduced within days. In accordance with inhibition of cell proliferation, the antagonists at short incubation periods (minutes-hours) induced key cell cycle regulators, p21, p27^40^ and cyclin D1^41^. To note, the antagonists effects were observed mainly at high molar concentrations. However, no toxicity was detected in mice treated with up to 60 mg/kg tetrac (equivalent to 250 μM tetrac concentration)^32^. Similarly, triac concentration in preclinical and clinical studies was reported to be in the range of 10–25 μM^36,42,43^, comparable with the range examined in this study. For T1AM, no toxicity was observed at 50 mg/kg^44^. The outcome of lower concentrations of tetrac and triac, but not T1AM, produced an agonistic effect on ovarian cancer cell number. This may be due to a phenomenon known as “ligand-induced-binding-site” (LIBS), in which natural or synthetic ligands may paradoxically induce rapid and potent clustering of the αvβ3 integrin on the cell surface. Therefore, low concentrations of antagonists may induce LIBS but will not be sufficient to occupy all integrin molecules and as such will activate, rather than inhibit, the integrin. A similar effect was documented with other integrin inhibitors, such as the cyclic RGD (e.g Cilengitide), for which mM concentrations are required to produce a beneficial therapeutic effect^45^.

After observing the anti-proliferative effect of the antagonists, we were interested in evaluating their effect on cell death. Our collective results show that tetrac produced cell death in the low grade ovarian cancer cells (A2780), but not in the high-grade cells. In contrast, triac and T1AM effectively induced cell death in both cell lines. In order to prove, regardless of cancer, that apoptosis activation by the derivatives requires direct interaction with an intact αvβ3 integrin, a platform of cells lacking or overexpressing the receptor was utilized. Using these cell models we were able to further highlight that αvβ3 integrin is required for triac and T1AM cytotoxic action.

In all cases, cell death initiated by the antagonists was reversed by the pan-caspase inhibitor, Z-VAD-FMK^46^, indicating a caspase-mediated mechanism. We have further validated the involvement of caspase-dependent cell death for all antagonists by measuring cleavage of caspase-3, a central effector caspase that is associated with the initiation of the ‘death cascade’^47^. In addition, cleavage of PARP-1, a downstream caspase-3 substrate^48,49^ that functions as a molecular marker for apoptosis^48^, was quantified. For the low-grade A2780 cells, caspase-3 activation followed by PARP-1 cleavage were clearly documented. However, no caspase-3 or PARP-1 cleavage was observed in the high-grade OVCAR3 cells following treatment with the various antagonists, suggesting the involvement of another effector caspase yet to be determined. As apoptotic cell death can also occur without activation of caspases, we considered a potential caspase-independent mechanism, namely the activation of apoptosis-inducing factor (AIF). This 67 kDa protein is cleaved by peptidase generating the mature 62 kDa protein^50^ and once inside the nucleus, participates in DNA fragmentation and chromatin condensation^51,52^. Our results indicate that all antagonists induced AIF in the low-grade ovarian cancer cells, which preceded the activation of caspase-3. AIF was reported before to act as a caspase-independent death effector that can function in parallel and independent of the mitochondrial activated caspase cascade^53^. However, no induction in AIF was documented in the high-grade cells. Consistently, the pro-apoptotic activity by the antagonists was less profound in the high-grade cells. This observation is not unexpected, as OVCAR3 cells, in accord with the drug resistance in high-grade ovarian cancer patients, are known to be highly resistant to an array of drugs, including adriamycin, cisplatin and melphalan^54^. A partial explanation for this death-resistance may be attributed to the *p53* status in these cells, a commonly mutated tumor suppressor gene in high-grade serous ovarian cancer^23,25^. OVCAR3 cells express the most common *p53* mutation in high-grade disease (R248W). This R248W *p53* mutation lacks normal p53 function and concomitantly gained novel oncogenic functions^55^, often with deleterious effects. A2780 cells, on the other hand, representing low-grade disease, carry a wild-type p53 protein^23,25^. Our results indicate that while A2780 cells were responsive to tetrac-induced apoptosis, OVCAR3 cells were completely resistant. This may suggest that an intact p53 is essential for tetrac’s action. In contrast to tetrac, triac and, more so T1AM, were highly effective in inducting apoptosis in both cell models, although a higher magnitude of apoptosis was observed in the low-grade p53-wild-type A2780 cells. It should be noted, however, that alterations in few pro-apoptotic genes were detected in the high-grade OVCAR3 cells in the presence of the three antagonists. Similar pro-apoptotic effects on gene transcription were previously reported for tetrac and its nanoparticulate form (known as nanotetrac or nano-diamino-tetrac) in other cancer models^28,29,31,56^ and are reviewed in^6,7^.

Lastly, DNA damage response by all three antagonists was studied. Two key proteins that coordinate recognition of DNA damage are ATM^57^ and PARP-1^58^. Induction of ATM phosphorylation and PARP-1 by the antagonists was shown in the ovarian cancer cells. Concordant with the higher apoptotic response, the low grade A2780 cells were more sensitive to DNA damage induction by tetrac than were the high grade OVCAR3 cells. However, it is interesting to note that earlier and more intense induction of DNA damage was documented in OVCAR3 by triac and T1AM. DNA damage by the three antagonists was shown to be indirect. We therefore suggest that these three agents do not directly bind to and induce DNA breaks, rather they may affect DNA repair mechanisms. This assumption is well supported for tetrac, by experiments in brain cancer cells in which this drug did not induce direct DNA damage but led to a potent reduction in repair mechanisms^59^.

In conclusion, our results demonstrate cytotoxic effects of three thyroid hormone derivatives, tetrac, triac and T1AM in ovarian cancer cells, with effects on cell proliferation, viability, apoptosis and DNA damage. Chemical modifications of triac and T1AM, may improve their performance, similar to positive results using the covalent nanoformulation of tetrac. Further studies are required to examine whether these novel derivatives may be harnessed and applied in the treatment of ovarian cancer.

## Methods

### Cell lines

Two ovarian cancer cell lines, representing type II high grade ovarian tumor (OVCAR3 cells, serous) and type I low grade tumor (A2780 cells, undefined histology) were used^23,25^. In parallel, the use of normal Chinese hamster ovary cell line (CHOK1) served as control cells. Another cell model which was utilized is HEK293 cell line (ATCC CRL1573) as well as HEK293, stably transfected with β3 integrin (A generous gift from Professor Jean-Luc Col, Institut Albert Bonniot and Universite’ Joseph Fourier, France). All cells were cultured in RPMI1640 supplemented with 10% heat-inactivated fetal bovine serum and antibiotics. β3-transfected HEK293 cells were grown in the presence of 700 μg/ml Geniticin (G418 sulfate, Gibco, Paisley, UK). All cell lines were authenticated and tested negative for mycoplasma contamination.

### Reagents and chemicals

Tetrac and triac were obtained from Sigma-Aldrich (Steinheim, Germany). T1AM was purchased from ABX, Radeberg, Germany. Z-VAD-FMK was purchased from Enzo life sciences (Laufen, Switzerland). For long incubation periods (days), the derivatives were added daily to existing media. Primary anti human antibodies against p21 (#2947), p27 (#3686), CycD1 (#2978), total/cleaved caspase 3 (#9665), PARP-1 (#9542) were from Cell Signaling technology (Leiden, The Netherlands). AIF (AB16501) and phycoerythrin (PE) conjugated αvβ3 antibody (clone LM609, MAB1976) were from Merck Millipore (Darmstadt, Germany). phospho ATM antibodies (phospho-serine1981, #2152-1) were from Epitomics (Burlingame, CA, USA). For protein loading normalization ponceau S solution (Sigma-Aldrich, Steinheim, Germany) or anti tubulin antibody (#2128, Cell Signaling technology) were used.

### Flow cytometry

(MACSQuant, Miltenyi Biotec, Bergisch Gladbach, Germany): For absolute cell number, the cells were harvested in a fixed volume and counted. For annexin-PI assay, cells were harvested and incubated with annexin V-FITC and PI (BioVision Inc., Milpitas, CA, USA) according to manufacturer’s instructions. Annexin−/PI−, surviving cell fraction; annexin +/PI−, early apoptosis; annexin +/PI+, late apoptosis and annexin−/PI+, late apoptosis/necrosis. For cell cycle analysis the cells were stained with PI, harvested and the different cell cycle phases (SubG1, Go/G1 and S-G2M) were gated and analyzed. For αvβ3 estimation, the cells were harvested in RPMI 1640 and labeled with 10 μg/ml PE-αvβ3 antibody (LM609).

### Viability assay

PrestoBlue cell viability reagent (10% final concentration, Invitrogen, Carlsbad. CA, USA) was incubated with cells at 37 °C for 0.5 hours and read with a microELISA reader at 570 nm excitation and 600 nm emission.

### Western blotting

Equal amounts of whole-cell lysates (15 μg) were separated on 10–12.5% polyacrylamid gels, transferred to polyvinylidene difluoride (PVDF) membranes, incubated with the indicated antibodies and visualized using horseradish peroxidase (HRP) conjugated secondary antibody (1:10 000, Jackson Immuno Research Laboratories, West Grove, PA, USA) followed by enhanced chemiluminescence detection (Biological Industries, Bet Haemek, Israel). Integrated optical densities of the bands were measured by Image reader Las3000, Multi-gauge v3.0 software. Optical densities were normalized to β tubulin or a general protein stain (ponceau).

### BrdU incorporation

BrdU (Exalpha Biologicals, Inc. Shirley, MA, USA) was added overnight to treated cells, after which the cells were fixed, permeabilized and the DNA denatured. An anti-BrdU monoclonal antibody was added for 1 hour, followed by the addition of HRP-conjugated goat anti-mouse antibody. The color reaction of the tetra-methylbenzidine product was quantified by a microELISA reader at 450/550 nm.

### RNA extraction and cDNA synthesis

RNA was extracted using NucleoSpine RNA II kit (Macherey-Nagel, Düren, Germany) according to the manufacturer’s instructions and eluted in 40 μL RNase free water. RNA concentration and purity were measured using NanoDrop™ 1000 Spectrophotometer (Thermo Scientific, Wilmington, DE, USA). RNA (200 ng) was reverse-transcribed using High Capacity cDNA Reverse Transcription Kit (Applied Biosystems, Carlsbad, CA, USA), according to manufacturer instructions.

### Real-time PCR

mRNA levels were measured by Real-Time PCR (7500 Fast system, Applied Biosystems, Carlsbad, CA, USA), using Taqman or Fast Sybr Green Master Mix (Applied Biosystems). Results were calculated as fold change using the comparative threshold cycle method (2-ΔΔCT) relative to control cells (that is, controls are arbitrarily assigned a value of 1). Taqman assays were used for *Bnip3L/Nix* (Hs00188949_m1) and *puma* (Hs00248075_m1). For the following targets, primers (Hylabs, Rehovot, Israel) were designed (Primer-Express software, Applied Biosystems) in different exons to minimize DNA contamination: *Apaf-1*: forward: TGCGCTGCTCTGCCTTCT. Reverse: CCATGGGTAGCAGCTCCTTCT. *NOXA* forward: CGGAGATGCC TGGGAAGAA. Reverse: CCAAATCTCCTGAGTTGAGTAGCA. *BAX* forward: TGGCAGCTGACATGTTTTCTG. Reverse: GGTGCACAGGGCCTTGAG. *Caspase-3* forward primer: CAGACAGTGGTGTTGATGATGAC. Reverse: TCGCCAAGAATAATAACCAGGTG.

### Microscopy

Cells were visualized by a fluorescence microscope equipped with a camera (model IX71; Olympus, Tokyo, Japan) with a × 20/0.50 objective lens and Cell^A (version 3.1) Olympus software imaging.

### DNA retardation assay

A cell-free experimental platform was utilized for DNA retardation assays^60^. This system examines the possible direct interaction of a chosen substance with the DNA molecule by a gel retardation assay, as shown by the disappearance of the DNA fragments from their normal positions. In addition, this assay provided information regarding the potential DNA shredding activity of the tested element, as shown by smearing of the DNA staining. For measuring direct DNA binding and breaks, linear DNA ladder (Thermo Scientific, Wilmington, DE, USA) and DNA extracted from OVCAR3 cells (DNA extraction kit, QIAGEN, Valencia, CA, USA) were incubated overnight in the presence or absence of a high dose of tetrac/ triac/T1AM (50 μM), after which DNA was separated by 4% agarose-gel electrophoresis.

### Statistical analysis

Experiments were analyzed by a Student’s unpaired *t* test for significance (p < 0.05).

### Data availability

All data generated or analysed during this study are included in this published article (and its Supplementary Information files).

## Electronic supplementary material

Supplementary Figures
