# Supplementary material for: Thyroid hormones derivatives reduce proliferation and induce cell death and DNA damage in ovarian cancer
Source: Sci Rep. 2017 Nov 28;7:16475. doi: 10.1038/s41598-017-16593-x (PMC5705594; doi:10.1038/s41598-017-16593-x)
Supplement: Supplementary file 1 — Supplementary Figures [file 41598_2017_16593_MOESM1_ESM.pdf]

Supplementary Materials:

Thyroid hormone derivatives reduce proliferation and induce cell death and DNA damage in ovarian cancer

Elena Shinderman-Maman[1,2,3], Keren Cohen[1,2,3], Dotan Moskovich[1,2,3], Aleck Hercbergs[4], Haim Werner[2,3], Paul J Davis[5], Martin Ellis[1,3] and Osnat Ashur-Fabian[1,2,3]

[1] Translational Hemato-Oncology Laboratory, The Hematology Institute and Blood Bank, Meir Medical Center, Kfar-Saba, Israel

[2] Department of Human Molecular Genetics and Biochemistry

[3] Sackler Faculty of Medicine, Tel Aviv University, Tel Aviv, Israel

[4] Radiation Oncology, Cleveland Clinic, Cleveland, OH, USA

[5] Department of Medicine, Albany Medical College, Albany, NY, USA

Correspondence: Dr. Osnat Ashur-Fabian, Translational Hemato-Oncology Laboratory, The Hematology Institute and Blood Bank, Meir Medical Center, Tchernichovsky 59, Kfar-Saba 6997801, Israel. E-mail: osnataf@gmail.com

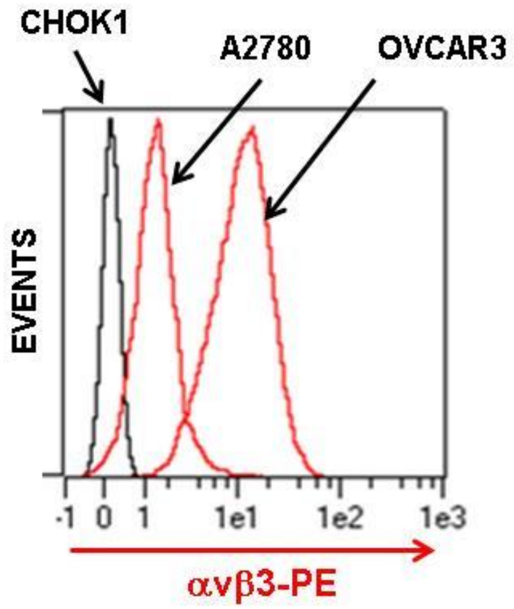

Supplementary Figure 1: OVCAR3, A2780 and CHOK1 cell lines were analyzed by flow-cytometry for αvβ3 expression

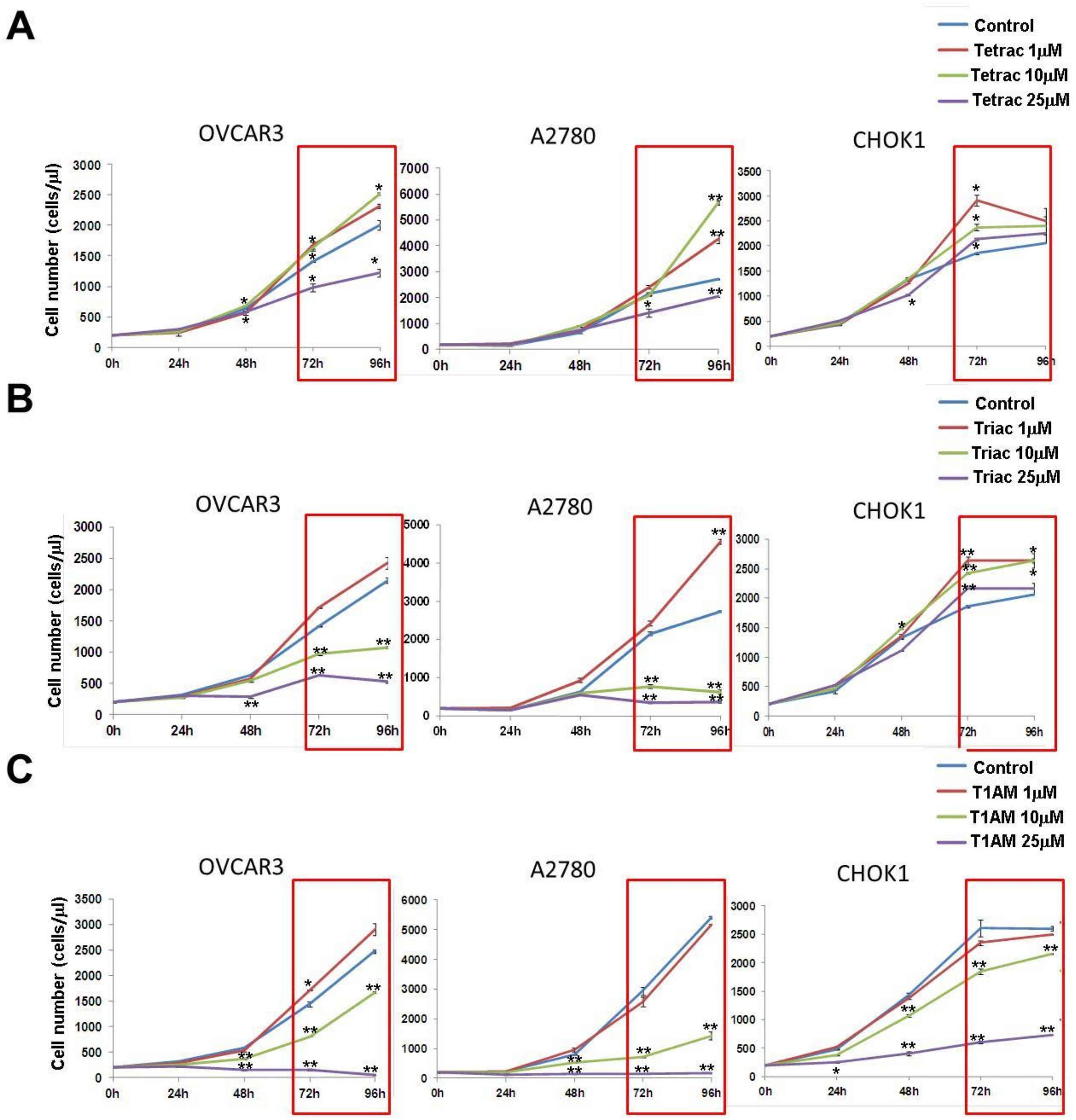

Supplementary Figure 2: Cells were treated with 1, 10 or 25 μM of **(A)** Tetrac **(B)** Triac or **(C)** T1AM for 24h-96h and cell number was evaluated using flow cytometry. Experiments were repeated three times in triplicates. Significance (* p<0.05, ** p<0.005) from vehicle control is shown.

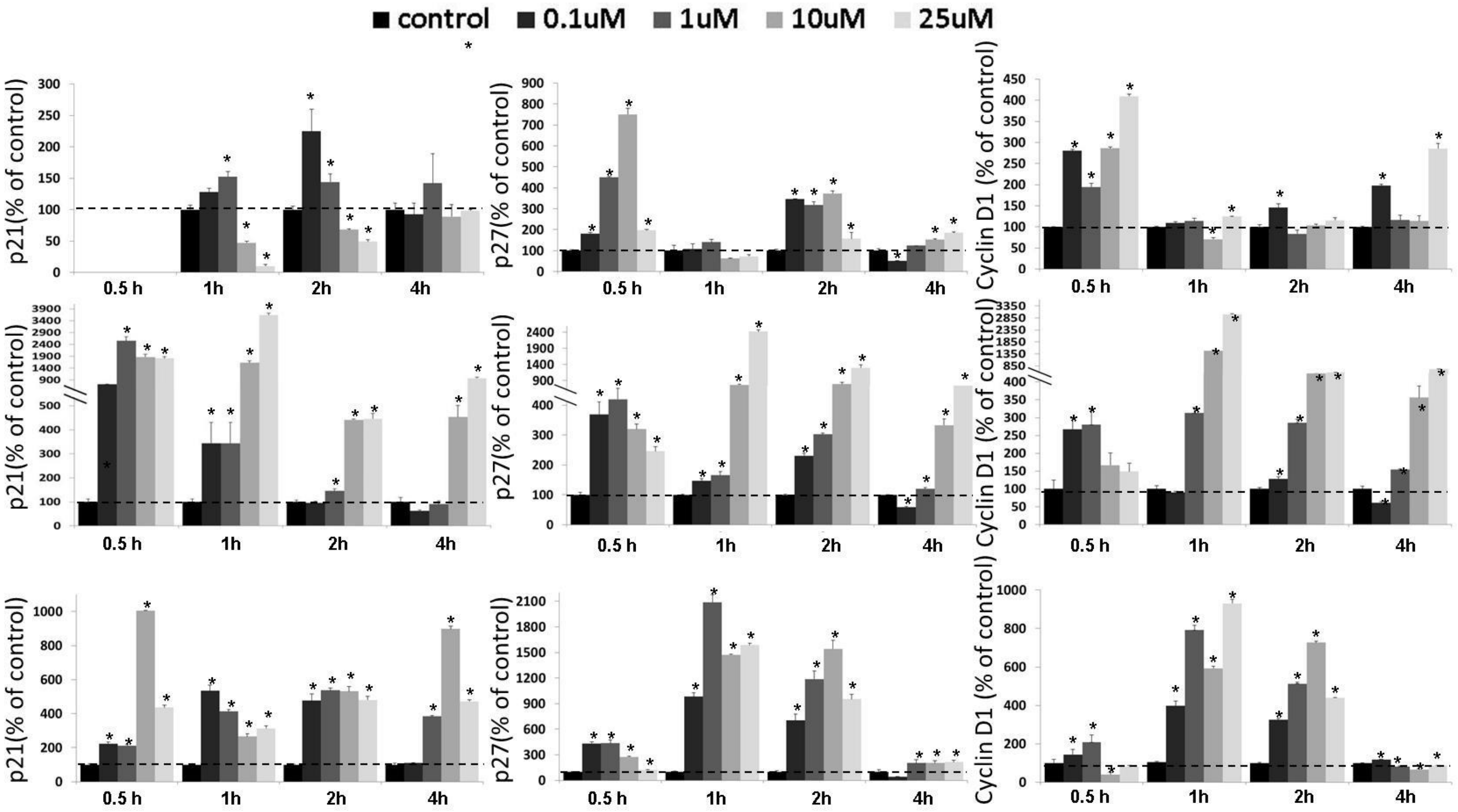

Supplementary Figure 3: Western blots quantification of p21, p27 and cyclin D1 protein levels (normalized to protein loading) following treatments with 0.1, 1, 10 or 25μM **(A)** tetrac **(B)** triac **(C)** T1AM for 0.5-4 hours in OVCAR3 cells.. * $p<0.05$.

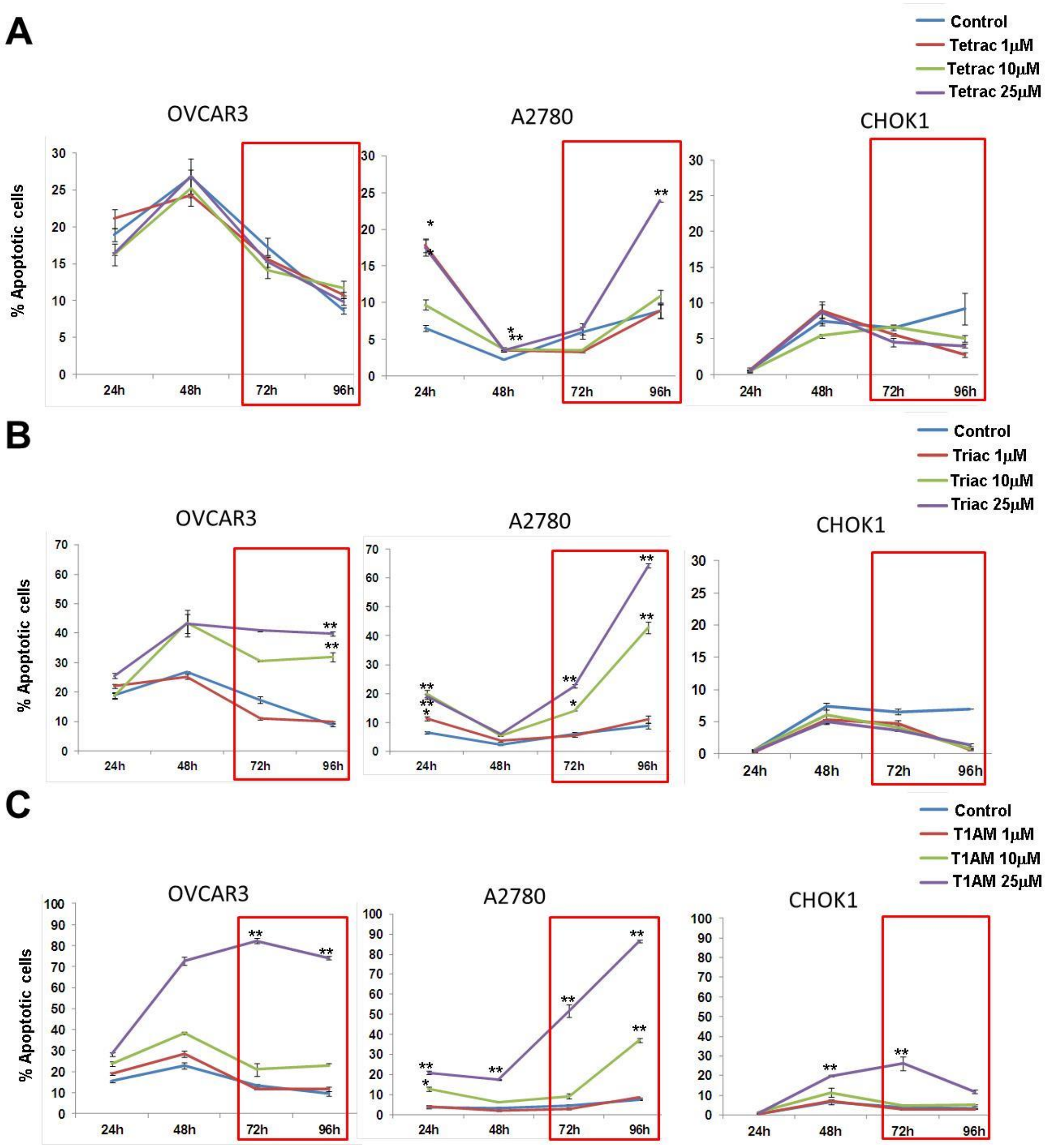

Supplementary Figure 4: Cells were treated with 1, 10 or 25 μM of **(A)** Tetrac **(B)** Triac or **(C)** T1AM for 24h-96h and analyzed for annexin-FITC/PI by flow-cytometry. Experiments were repeated three times in triplicates. Significance (* $p<0.05$, ** $p<0.005$) from vehicle control is shown.

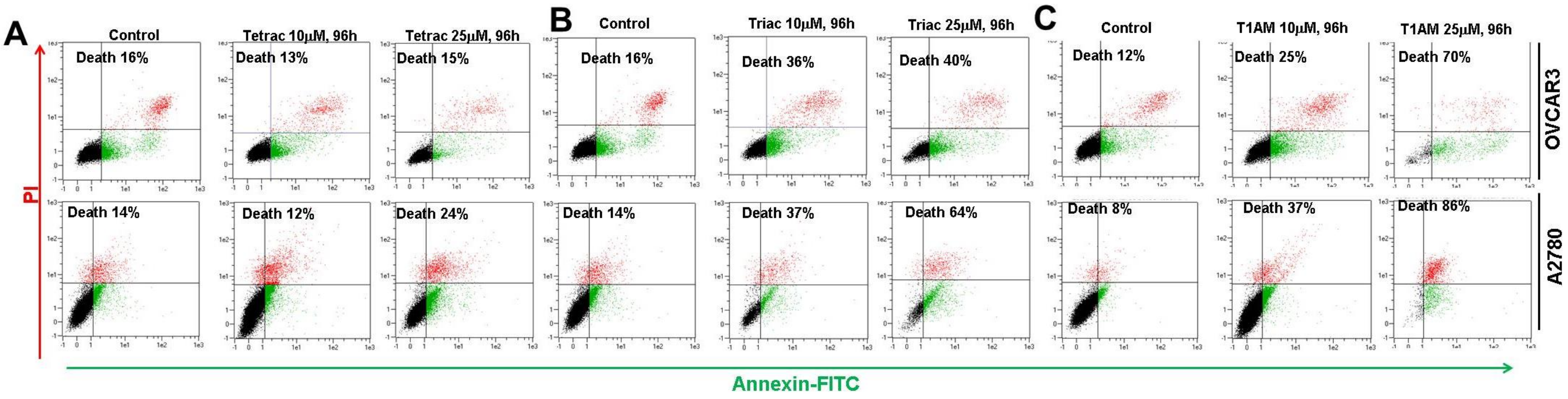

Supplementary Figure 5: Cells were treated with 10 or 25 µM of **(A)** Tetrac **(B)** Triac or **(C)** T1AM for 96h and and analyzed for annexin-FITC/PI. Flow cytometry plots from representative experiments are shown.

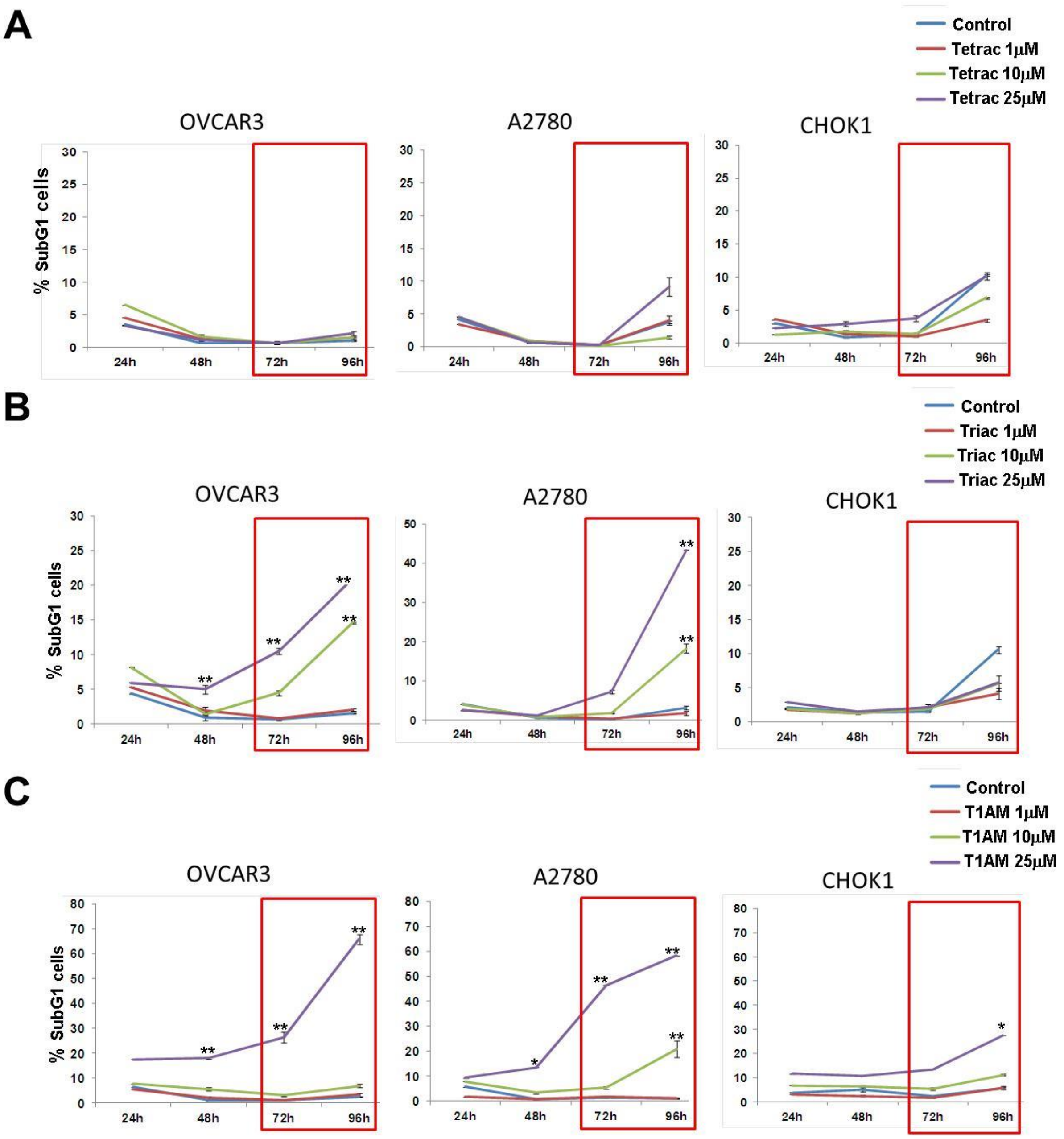

Supplementary Figure 6: Cells were treated with 1, 10 or 25 µM of **(A)** Tetrac **(B)** Triac or **(C)** T1AM for 24h-96h, cell cycle analysis was performed and the changes in subG1 cell fraction over time were documented. Experiments were repeated three times in triplicates. Significance (* p<0.05, ** p<0.005) from vehicle control is shown.

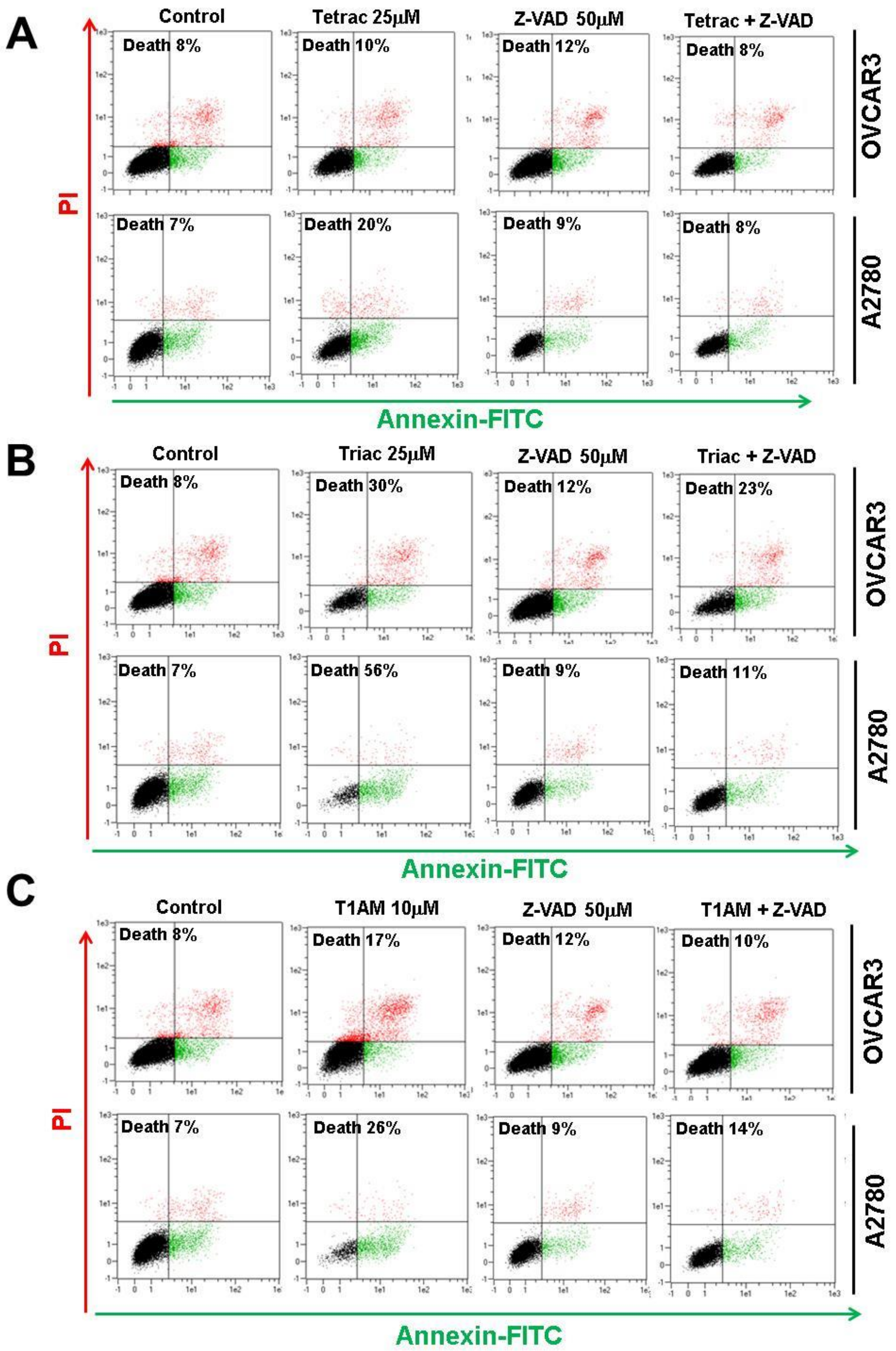

Supplementary Figure 7: Cells were treated with 25 µM **(A)** tetrac **(B)** triac **(C)** 10µM T1AM, for 96 hours ± Z-VAD-FMK (50 µM) and analyzed by annexin-V/PI. Experiments were repeated twice in triplicates. Representative Flow cytometry plots are presented.

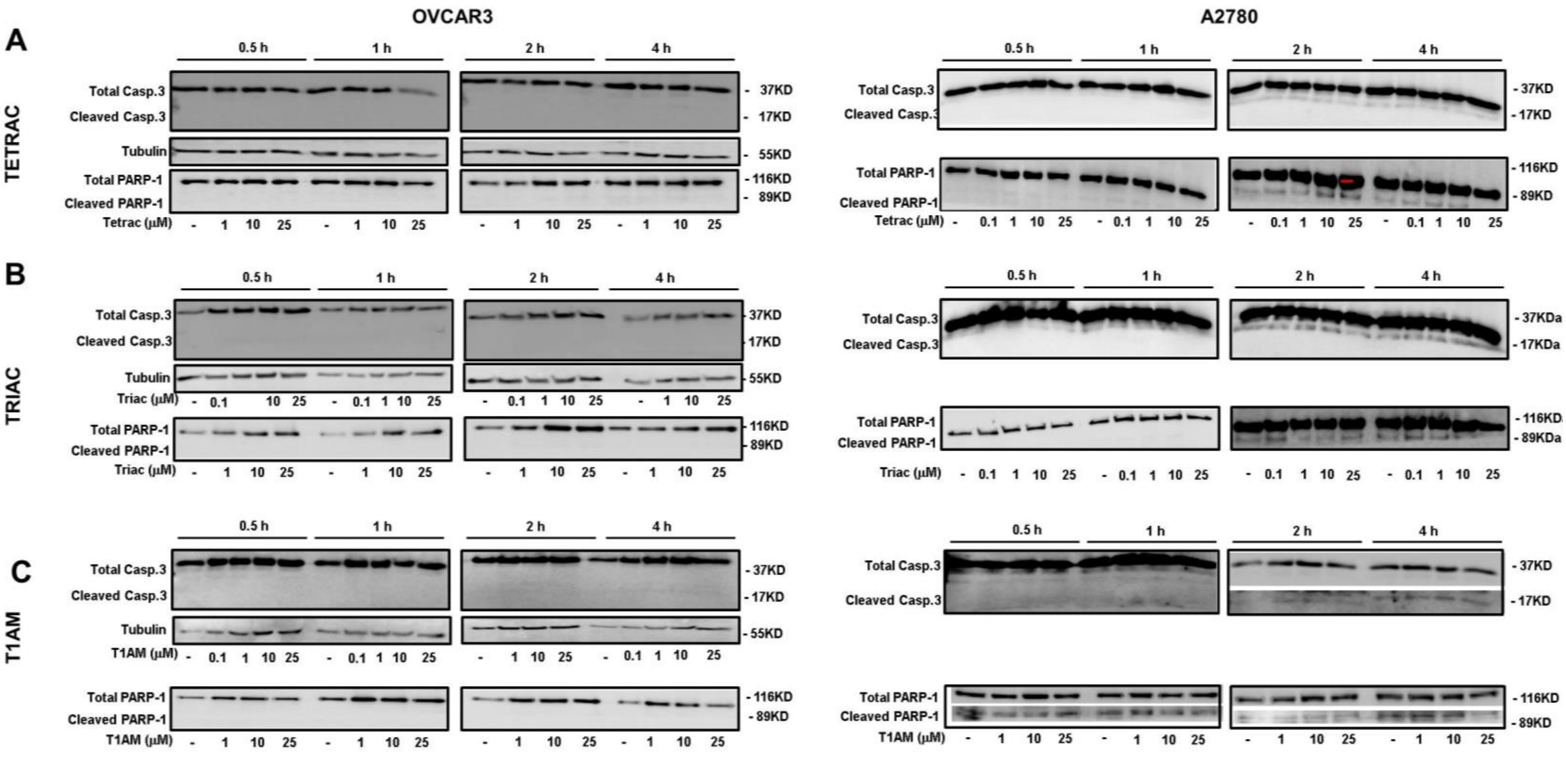

Supplementary Figure 8: Cells were treated with 0.1, 1, 10 or 25 μM **(A)** tetrac **(B)** triac or **(C)** T1AM, for 0.5-4 hours and caspase-3 and PARP-1 cleavage were quantified by Western blots. Representative blot is presented. Experiments were repeated twice.

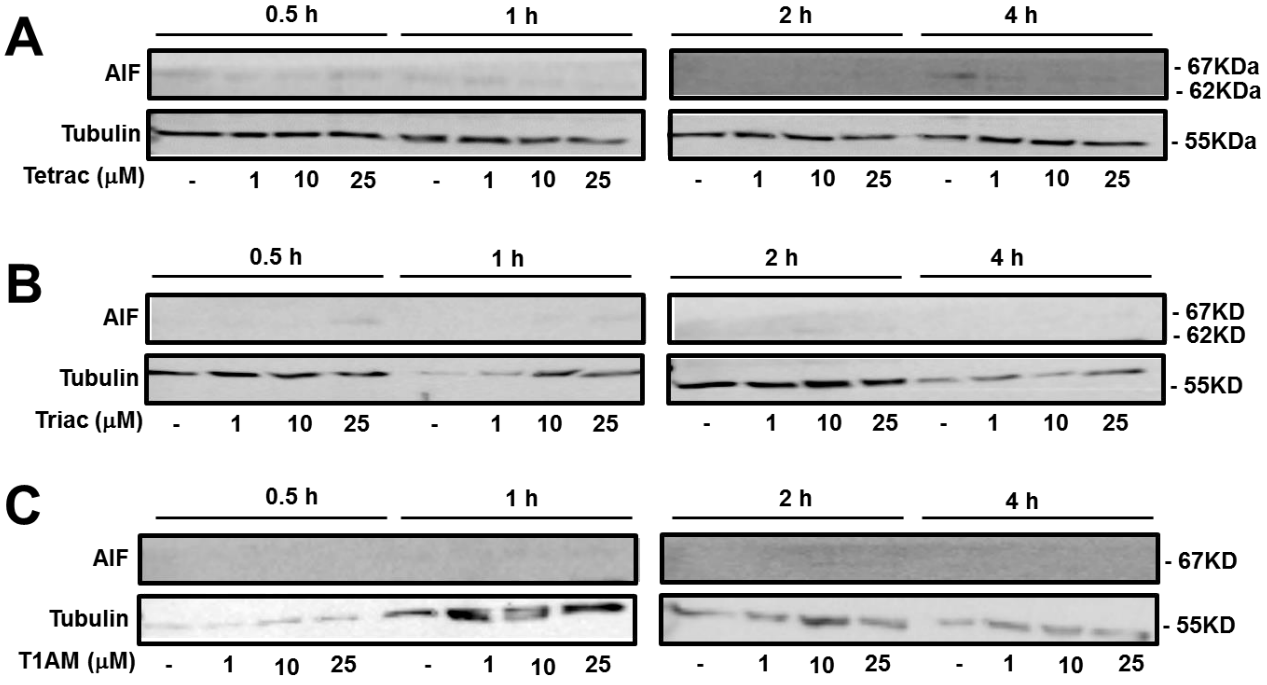

Supplementary Figure 9: OVCAR3 cells were treated with 1, 10 or 25μM **(A)** tetrac **(B)** triac **(C)** T1AM for 0.5-4 hours and AIF levels were evaluated by Western blots. Representative blot of duplicates is presented.

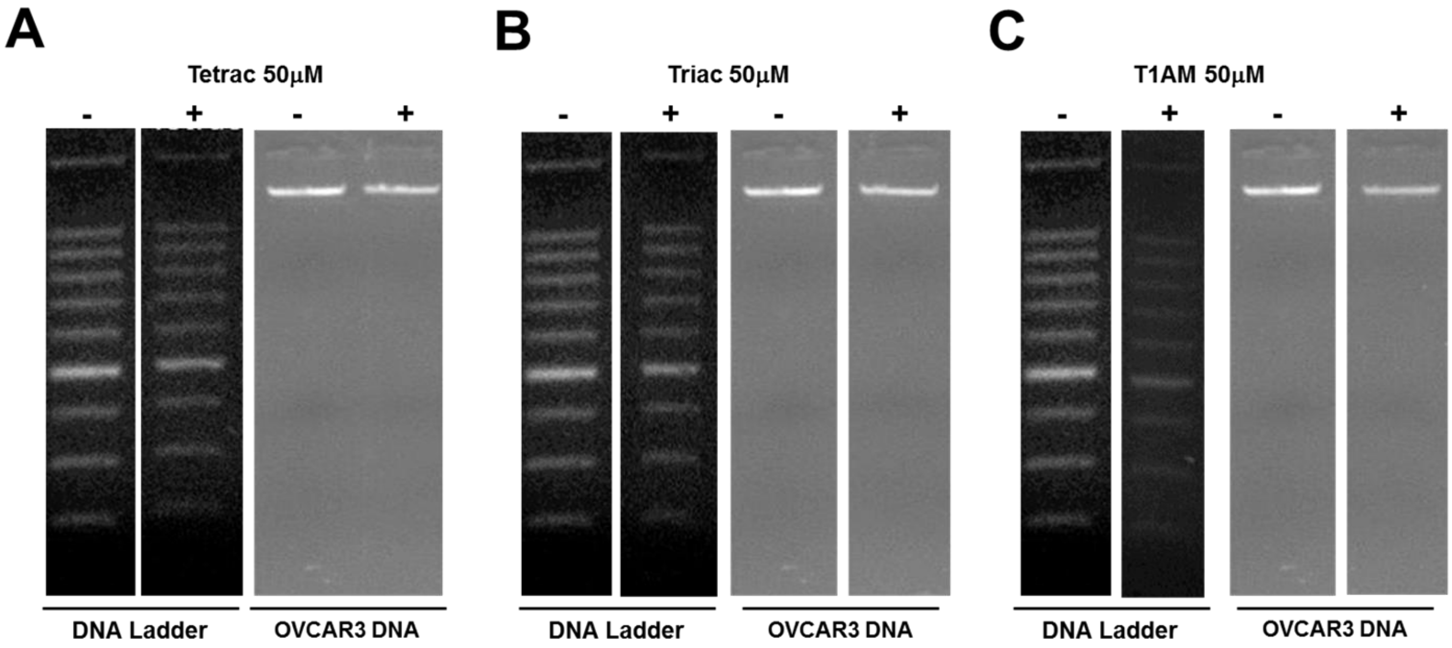

Supplementary Figure 10: Linear DNA ladder (left panels) and DNA extracted from OVCAR3 cells (right panels) were treated with 50 μM of **(A)** tetrac **(B)** triac or **(C)** T1AM for an overnight and separated by agarose gel. Experiments were repeated twice.
